# The aging lung: microenvironment, mechanisms, and diseases

**DOI:** 10.3389/fimmu.2024.1383503

**Published:** 2024-05-02

**Authors:** Yanmei Wang, Xuewen Huang, Guofeng Luo, Yunying Xu, Xiqian Deng, Yumeng Lin, Zhanzhan Wang, Shuwei Zhou, Siyu Wang, Haoran Chen, Tao Tao, Lei He, Luchuan Yang, Li Yang, Yutong Chen, Zi Jin, Chengshi He, Zhongyu Han, Xiaohong Zhang

**Affiliations:** ^1^ School of Medical and Life Sciences, Chengdu University of Traditional Chinese Medicine, Chengdu, China; ^2^ Institute of Traditional Chinese Medicine of Sichuan Academy of Chinese Medicine Sciences (Sichuan Second Hospital of T.C.M), Chengdu, China; ^3^ Eye School of Chengdu University of Traditional Chinese Medicine, Chengdu, China; ^4^ Department of Respiratory and Critical Care Medicine, The First People’s Hospital of Lianyungang, Lianyungang, China; ^5^ Jiangsu Key Laboratory of Molecular and Functional Imaging, Department of Radiology, Zhongda Hospital, School of Medicine, Southeast University, Nanjing, China; ^6^ Department of Gastroenterology, The First Hospital of Hunan University of Chinese Medicine, Changsha, China; ^7^ The Second Clinical Medical College, Zhejiang Chinese Medical University, Hangzhou, China; ^8^ Department of Anesthesiology and Pain Rehabilitation, Shanghai YangZhi Rehabilitation Hospital (Shanghai Sunshine Rehabilitation Center), School of Medicine, Tongji University, Shanghai, China; ^9^ Department of Respiratory, Hospital of Chengdu University of Traditional Chinese Medicine, Chengdu, China; ^10^ Department of Emergency Medicine Center, Sichuan Province People’s Hospital University of Electronic Science and Technology of China, Chengdu, China

**Keywords:** aging, immunity microenvironment, mechanism, lung diseases, therapy

## Abstract

With the development of global social economy and the deepening of the aging population, diseases related to aging have received increasing attention. The pathogenesis of many respiratory diseases remains unclear, and lung aging is an independent risk factor for respiratory diseases. The aging mechanism of the lung may be involved in the occurrence and development of respiratory diseases. Aging-induced immune, oxidative stress, inflammation, and telomere changes can directly induce and promote the occurrence and development of lung aging. Meanwhile, the occurrence of lung aging also further aggravates the immune stress and inflammatory response of respiratory diseases; the two mutually affect each other and promote the development of respiratory diseases. Explaining the mechanism and treatment direction of these respiratory diseases from the perspective of lung aging will be a new idea and research field. This review summarizes the changes in pulmonary microenvironment, metabolic mechanisms, and the progression of respiratory diseases associated with aging.

## Introduction

Aging is the gradual decline of physiological functions, characterized by signs like dry skin, wrinkles, and memory loss. Over the last two centuries, life expectancy in developed countries has doubled (1, 2). By 2050, it is expected that those over 65 will make up approximately 20% of the global population (3). Since 1939, caloric restriction in mice has been shown to extend lifespan (4). New research finds that targeting specific loci or altering metabolic pathways can delay the aging process (5).

Cellular senescence, a hallmark of aging, is the irreversible cessation of cell division, often marked by DNA damage, inflammatory secretions, and metabolic changes. It occurs during both development and adulthood, contributing to aging organ degeneration (6). However, the exact relationship between cellular senescence and aging, and how the former influences the latter, remains unclear.

The lung, crucial for gas exchange and sensitive to external stimuli, is highly susceptible to aging. It matures by age 10–12, reaching peak function at 20 in women and 25 in men (7). Aging degrades lung barrier integrity and pathogen resistance while increasing immune sensitivity, thus heightening disease risk and mortality from conditions like lung cancer and inflammation. Understanding how aging affects lung health and exacerbates pathological damage is a critical research area.

In this manuscript, we review the research progress on the role of pulmonary aging in the pathogenesis of respiratory diseases, including the mechanism and response pathway of how various cells in the pulmonary microenvironment cope with the molecular level of aging. We believe that this manuscript will help readers to further understand age-related respiratory diseases and can also provide new ideas for the study of the pathogenesis and clinical treatment of various respiratory diseases.

## Morphology, respiratory indices, and function changes in the aging lung

### Morphology changes

“Senile lung” describes lung aging, marked by alveolar and duct expansion, and basement membrane thickening, leading to decreased lung elasticity and increased compliance (2, 8).

Aging leads to terminal air space enlargement, increased alveolar duct and terminal bronchiole size, and a reduction in alveolar area and number. For instance, the total alveolar area decreases from 70 m^2^ in individuals aged 30–39 to approximately 60 m^2^ in those 70–79 years old, dropping approximately 2.5 m^2^ every decade (8, 9). Additionally, aging increases lung collagen content and thickens the alveolar basement membrane (10). These anatomical changes result in several physiological alterations: reduced elastic recoil, increased lung compliance, diminished oxygen diffusion capacity, early airway collapse, intrapulmonary gas retention, and decreased expiratory flowrate. These changes can obstruct small airways, potentially leading to chronic obstructive pulmonary disease (COPD) in the elderly (11). Furthermore, smoking exacerbates COPD risk and progression in this age group, intertwining with aging to accelerate the disease’s severity.

### Respiratory indices changes

After birth, total lung capacity (TLC) increases, but in the elderly, inspiratory muscle strength, thoracic compliance, and lung elastic recoil decrease, leading to lower TLC, tidal volume (TV), inspiratory reserve volume (IRV), and expiratory reserve volume (ERV) compared to young adults.

Vital capacity (VC), the maximum air volume exhaled after forced inspiration, decreases with age, approximately 200 ml every decade starting from age 20, from approximately 3,500 ml in men and 2,500 ml in women at age 30, to 75% of that by age 70 due to increased thoracic rigidity and decreased lung recoil and respiratory muscle strength.

Functional residual capacity (FRC), the gas volume remaining in the lungs after a quiet expiration, increases with age, leading to alveolar distension and increased respiratory muscle load. This results in a 20% increase in energy expenditure for respiration in 60-year-olds compared to 20-year-olds (12). Aging also enlarges alveoli and alters supportive structures, reducing lung elasticity and causing premature small airway closure during expiration, thus increasing FRC (12, 13).

The spirometric index FEV1/FVC, crucial for diagnosing COPD, shows that FEV1 and FVC peak at 27 years in men and 20 in women, then decline, with FEV1 decreasing faster after 65 years of age (14).

In addition, as respiratory indicators reflecting small airway ventilation function, expiratory flowrates (V25–75, Vmax25, Vmax75) decrease with age, indicating small airway function decline. Closed volume (CV)/VC and closed capacity (CC)/TLC ratios increase with age due to earlier small airway closure in the elderly during expiration.

### Function changes

Lung functionality peaks between ages 18 and 25 and remains stable until approximately 35 years old, after which it gradually declines (15). This decline is attributed to reduced respiratory muscle strength and chest wall function in older adults, leading to decreased ventilation capacity. In general, with increasing age, TV may slightly decrease. This is mainly due to the slight decrease in lung capacity, increased stiffness of lung tissue, and potential decline in respiratory muscle function as individuals age, all of which can impact TV. On the other hand, research has shown that TV can be increased through training, depending on individual lifestyle and physiological conditions (16).

From age 35, lung function decreases even in healthy individuals, with stiffer blood vessel walls and reduced elasticity impacting blood supply to alveoli and gas exchange (17–21). Aging also reduces alveolar surface area and capillary density, affecting lung ventilation function, all leading to ventilation/perfusion ratio imbalance and lower arterial oxygen partial pressure (PaO_2_) in the elderly (16). In older lungs, there is a diminished response to hypoxemia and hypercapnia and less recognition of bronchoconstriction (22). The aging lung’s response to hypoxemia and hypercapnia, measured by oral occlusion pressure, shows a 50% and 60% decrease, respectively, in the elderly compared to young adults, indicating a reduced ability to integrate sensory information and generate appropriate neural responses (21).

## Microenvironment of aging lung

For air-breathing animals, the lung is a vital organ of the respiratory system. Mammals and other structurally complex animals have two lungs, located on the left and right sides of the chest cavity near the spine and heart. The primary function of the lungs is to facilitate gas exchange, transporting oxygen from the air into the bloodstream and removing carbon dioxide from the blood into the atmosphere. As a functionally important and structurally complex organ, the aging lung harbors various types of cells, including resident cells and immune cells within the lung microenvironment. Previous research has utilized light microscopy and electron microscopy to observe the immune reactions and cellular morphology of lung tissue, defining resident cells in the lung (23). Advanced techniques such as single-cell sequencing and immunohistochemistry have expanded our understanding, revealing over 40 distinct cell types in human lungs. However, the effects of aging on lung cell composition and function remain partially understood. Age alone is a risk factor for lung diseases, with cellular aging contributing to different pathological outcomes. This knowledge opens new avenues for investigating chronic respiratory disease mechanisms by examining the aging lung’s unique structures and the biological impact of aging on different cell types.

## Lung parenchymal cells

“Lung parenchymal cells” refers to the cells that make up the functional tissue of the lungs. Comprising a multitude of minute alveoli with thin walls, it constitutes an immense surface area (24). These cells include various types, such as alveolar epithelial cells, bronchial epithelial cells, and endothelial cells. These cells each have their own functions; alveolar epithelial cells, for example, play a crucial role in gas exchange by lining the inner surface of the alveoli, where oxygen is taken up and carbon dioxide is released (25).

### Airway epithelial cells

Airways are divided into respiratory and conducting zones by terminal bronchioles. They secrete mucins and fluids, lined with respiratory cells and ciliated pseudostratified epithelium. Epithelial cells form polarized junctional complexes with claudins for protection (26). Ciliated cells are predominant, supported by club, serous, neuroendocrine, and goblet cells. Subepithelial basal cells serve as progenitors for airway epithelium regeneration (27). In the airways, submucosal glands secrete mucins (like MUC2 and MUC5B) and fluids and can release defense proteins upon stimulation (27, 28). Mucus, a high molecular weight glycoprotein, facilitates pathogen clearance and plays a role in maintaining homeostasis in the airway (29).

Aging can induce alterations in the function and structure of tracheal epithelial cells, characterized by diminished proliferative capacity, elevated apoptosis, and decreased metabolic activity. These modifications will impair tracheal epithelial cell function, consequently impacting the normal physiological function of the trachea.

### Alveolar epithelial cell

Alveolar epithelial cells (AECs), including squamous AEC1s and cuboidal AEC2s, are crucial for gas exchange. AEC1s cover approximately 95% of the respiratory membrane, while AEC2s, also progenitor cells for AEC1s, contribute to repair and innate immunity by releasing surfactants (30).

Experimental studies comparing young (2–3-months-old) and aged (26-months-old) rats revealed a decrease in alveolar epithelial cell (AEC) proliferation and surfactant protein levels in older rats, alongside an increase in apoptosis rate. Furthermore, an electron microscopy of aged lungs showed significant degenerative changes in AEC2s, with shorter telomere mice displaying cellular senescence markers like inflammation and immune responses (31). This senescence leads to prolonged oxidative stress and inflammation, impairing gas exchange across the alveolar membrane (32). Additionally, single-cell transcriptional analysis indicated an upregulation of MHC class I on aged AEC2s, highlighting aging’s impact on immune responses (33).

### Endothelial cell

Endothelial cells line the inner walls of blood vessels, forming a single layer crucial for maintaining vascular health through tight junctions and adhesion molecules like vascular endothelial cadherin (34, 35). They regulate blood vessel tone, permeability, and inflammation, playing a key role in vasodilation via nitric oxide (NO) production (36). Aging impairs these functions, leading to decreased NO synthesis and disrupted vascular relaxation, affecting molecules such as ICAM-1 and PAI-1 that are involved in inflammation and thrombosis, thereby increasing the risk of atherosclerosis and respiratory diseases (37). While insights are mainly from rodent studies, more research is needed to fully understand these mechanisms.

### Airway smooth muscle

Airway smooth muscle (ASM) plays a pivotal role in airway size regulation, utilizing adhesion molecules like E-cadherin and VCAM1 for stability and inflammation prevention (38). It interacts with extracellular matrix components via integrins to modulate contractility (39). Desmin, a cytoskeletal protein, is vital for cell shape, intracellular transport, and organelle organization, with its expression decreasing in aged lungs, potentially reducing airway contractility (40, 41). Conversely, the expression of alpha-smooth muscle actin and vimentin might increase in the distal airways of elderly lungs, indicating possibly enhanced peripheral airway contractility (41). These findings highlight complex age-related alterations in ASM function that warrant further investigation.

### Pulmonary progenitor cells

Pulmonary progenitor cells, vital for lung development and repair, differentiate into cell types like alveolar and capillary endothelial cells. Found in alveolar and airway regions, AT2 cells are key alveolar progenitors, transforming into AT1 cells for gas exchange upon stimulation (42). Research reveals two AT2 subtypes: surfactant producing and AT1 differentiating (43). Airway basal progenitor cells ensure lung stability and repair, generating various lung cells post-damage (44, 45). Interstitial progenitor cells, or fibroblasts, contribute to alveolar remodeling. Aging decreases stem cell numbers and repair efficiency, leading to diseases like emphysema and pulmonary fibrosis (46). The effect of aging on lung stem cells and remodeling needs further exploration. Understanding how aging impacts airway function is vital for improving elderly health.

## Interstitial region of the lung

The lung interstitium, situated between lung parenchyma, consists of connective tissue, lymphatics, nerve fibers, and blood vessels, crucial for structure, nutrition, and gas exchange support. It encompasses the central and peripheral fibrous systems and septal tissue, essential for maintaining alveolar-capillary gas exchange integrity.

Fibroblasts are an important component of the interstitial region, generating extracellular matrix (ECM) components like collagen fibers, and matrix metalloproteinases (MMPs) play a key role in tissue integrity. Aging fibroblasts are implicated in lung remodeling and respiratory diseases (47). To understand the changes in fibroblasts under the influence of aging, several research groups have shown altered ECM protein expression, as evidenced by proteomics and microarray studies (48). Additionally, single-cell RNA sequencing indicates decreased collagen XIV and decorin in aged fibroblasts, affecting the lung tissue’s integrity and elasticity (49). Researchers have utilized methods like microarray, liquid chromatography-mass spectrometry, and atomic force microscopy to study the ECM in the lung and its relation to aging (49–52). They identified at least 32 age-related proteins in the lung’s ECM, whose changes disrupt its biomechanical balance, leading to aging-related damage in lung tissues.

The pulmonary microenvironment represents a complex ecosystem, wherein each component plays a pivotal role in the aging process. When exploring the impact of the pulmonary microenvironment on health, an unavoidable question emerges: does cellular senescence constitute the core driving force behind pulmonary aging? This question is thought-provoking, as it is closely related to the decline in lung function and the onset and progression of pulmonary diseases. Cellular senescence is a multifactorial-driven process, involving alterations in gene expression. In the lungs, this process may be accelerated by factors such as environmental pollutants, smoking, and chronic inflammation. Therefore, understanding how cellular senescence affects the pulmonary microenvironment, and how to intervene in this process to decelerate pulmonary aging, has become a focal point of current research.

## Immunity and inflammation in aging lung

Aging significantly impacts pulmonary immunity by affecting immune cells in the lungs. Alveolar macrophages (AMs), crucial for innate immunity, show altered cytokine secretion and reduced phagocytosis abilities with age, leading to slower immune responses (53, 54). Other immune cells also experience quantitative and functional declines, affecting monocyte production and T- and B-cell receptor expression, ultimately compromising lymphocyte function (55). Research suggests that the aging phenotype of circulating monocytes is influenced by the pulmonary microenvironment, highlighting the role of the aging microenvironment in immune function changes (56). Immune senescence in the elderly increases susceptibility to infections and lung diseases, emphasizing the importance of understanding age-related changes for improving respiratory disease outcomes in older individuals.

### Innate immunity

Innate immunity, our first line of non-specific defense present from birth, includes barriers like skin and internal components such as phagocytes (e.g., neutrophils, macrophages) and natural killer cells. These elements identify and fight off pathogens, triggering inflammation for pathogen removal and tissue healing. However, aging can weaken these immune cells, disturbing the balance of inflammatory responses in the lungs. This imbalance exacerbates outcomes in elderly patients with inflammatory lung conditions. This section delves into the primary innate immune cells in the lungs and how aging affects their functionality.

#### Alveolar macrophages

AMs, part of the mononuclear-phagocyte system, are long-lived and numerous, playing critical roles in pulmonary immunity by collaborating with bronchial perivascular interstitial macrophages (IMs) and pulmonary epithelial cells (57). They clear debris and toxic particles, produce anti-inflammatory factors like IL-4 and IL-10, and are key in tissue damage control and initiating inflammatory responses (58, 59). Additionally, AMs recognize stimuli through PRRs, activating signaling pathways and cytokine release (e.g., TNF-α, IL-6) from epithelial cells, thus recruiting immune cells and promoting inflammation. Impaired AM function can lead to chronic inflammation or fibrosis due to the accumulation of activated AMs and excessive immune cell recruitment.

With aging, the decline in AM number and functionality impairs pulmonary innate immunity, increasing susceptibility to chronic inflammatory lung diseases in the elderly (60). Aged AMs exhibit weakened phagocytosis and pathogen clearance, reduced lipid breakdown, and increased lipoprotein deposition in alveoli (60–62). Moreover, aged macrophages produce fewer chemokines and cytokines, weakening the innate immune response (63). Age-related changes in cell communication and PRRs expression heighten vulnerability to infections (64). Elevated reactive oxygen species (ROS) levels with age further diminish AM function (65, 66). Consequently, reduced AM efficacy leads to heightened lung inflammation and tissue damage in the elderly.

#### Dendritic cells

Dendritic cells (DCs) are located in the alveoli, alveolar septa, and lung lymphatic tissues (67). They play a key role in antigen presentation and immune regulation. Despite similar morphologies between young and aged DCs, upon encountering foreign antigens, DCs utilize their dendritic projections to capture and internalize these antigens. Following internalization, antigens are processed and presented on the DC surface as antigen–protein complexes via MHC molecules. Stimulated by PRRs, DCs produce cytokines like TNF-α and IL-6, and mature DCs migrate to pulmonary lymph nodes to present antigen information to T cells, facilitating their differentiation into effector or memory T cells. This antigen presentation process is vital for immune response regulation.

In summary, DCs play a vital role in lung immune responses, but their number and function decline with age, leading to reduced antigen capture and processing abilities (68, 69). Therefore, these changes contribute to raise the risk of respiratory diseases potentially.

#### Innate lymphocytes

Innate lymphocytes, categorized into ILC1, ILC2, ILC3, and NK cells, are pivotal in immune defense. NK cells, part of Group 1 with ILC1, are notable for their capacity to eliminate tumor and infected cells by detecting changes like the absence of MHC-I molecules on the cell surface and by secreting cytotoxins (e.g., perforin) and cytokines (IFN-γ and TNF-α) (70). Their decline with age increases the risk of lung diseases in the elderly by impairing immune functions (71). ILC2 cells, through IL4 and IL5 secretion, target extracellular pathogens and allergens, while ILC3 cells, producing IL17 and IL22, aid in lymph node development. Collectively, ILCs are crucial in pulmonary health and innate immune system regulation.

#### Neutrophils

Neutrophils, comprising 50%–70% of white blood cells, are essential for the immune response, rapidly migrating to infection sites via chemotaxis and utilizing lysosomal enzymes to digest pathogens and debris, thus preventing infection spread (72). They also recruit additional immune cells by releasing inflammatory mediators. However, aging leads to decreased bone marrow production and reduced neutrophil counts, alongside diminished antioxidant capacity and increased ROS production, impairing phagocytosis and heightening infection risks (73–75). Studies indicate an age-related increase in neutrophils within bronchoalveolar lavage fluid (BAL) and imbalances in injury models (75, 76). Therefore, we can conclude that aging and injury prolong neutrophil recruitment times, causing accumulation in lung tissue and exacerbating pulmonary diseases and inflammation (76–78).

## Adaptive immunity

Adaptive immunity combats foreign pathogens through specificity, memory, cell dependence, and clone selectivity. It targets specific pathogens via receptors on B and T cells. Memory allows for a rapid response upon re-exposure to the same pathogen, facilitated by long-lasting immune cells generated after initial contact. Clone selectivity, through diverse B- and T-cell clones, ensures effective, enduring protection against various pathogens.

Bronchus-associated lymphoid tissue (BALT) is a lymphoid tissue present beneath the respiratory mucosal layer, including lymph nodes, lymphoid follicles, and diffuse lymphoid tissue, playing a role in immune surveillance and defense, thereby protecting the respiratory system from infection and disease.

### T lymphocytes

T lymphocytes, critical for cell-mediated immunity, originate as precursor cells in the bone marrow and mature into naive T cells in the thymus, expressing CD4+ or CD8+ for antigen recognition. In the elderly, naive T-cell production and TCR diversity decline due to miR181a deficiency and increased dual-specific phosphatase(DUSP)6 activity (79). These cells, upon antigen exposure and cytokine activation (e.g., IL-2, IL-4), differentiate into effector and memory T cells, with effector cells being either helper T cells (Th) releasing cytokines to modulate immune responses or cytotoxic T cells (CTL) that eliminate infected or cancerous cells through perforin and granzyme B. CD4+ T cells diversify into Th1, Th2, and Th17 based on cytokine profile, while Th17 and Treg cells share a precursor requiring TGF-β for differentiation (80). Aging impacts T-cell quantity and functionality, manifesting as reduced CD8+ T-cell proliferation, increased apoptosis susceptibility in CD4+ cells due to elevated CD39 expression, and diminished pathogen clearance, leading to compromised pulmonary immunity (81). This contributes to immune senescence, characterized by a delayed response to new antigens and inefficient immune memory formation.

### B lymphocytes

B-cell development initiates in the bone marrow from hematopoietic stem cells, requiring BCR ligand binding for progression. Immature B cells evolve through T1 and T2 transitional stages. Driven by CXCL13 and CXCR5, they migrate to the spleen, becoming T1B cells and further mature into T2B cells (82). These T2B cells differentiate into either follicular or marginal zone cells based on receptor signals (83). Naive B cells, which have not encountered antigens, include all spleen-resident B cells. Upon injury and inflammation, B cells activate, producing plasma cells that secrete antibodies, including immunoglobulins (Ig) and complement, and memory B cells for sustained immune memory against antigens, which protects the human body (84). Age affects B-cell development, particularly from naive to mature stages, with elderly mice showing increased inhibitory TFR cell expansion, fewer initial and immature B cells, and reduced antibody specificity and affinity, raising the risk of lung diseases in the elderly (85, 86).

Although there have been many studies on age-related changes in innate and adaptive immunity, the question of how immune impairment leads to lung diseases and increases mortality risk still remains. The combination of individual genetics and environmental changes still brings us many unknowns and challenges. To solve this problem, multi-omics methods have been used to longitudinally describe individual immune systems, which has also facilitated the development of “immune aging” scores that better describe an individual’s immune state than their actual age (87). This study and others have emphasized an important concept that actual age is not a reliable indicator of biological age.

## Senescence mechanism related to pulmonary disease

Aging can lead to a decrease in the number and functional defects of lung stem cells, and pulmonary remodeling. One of the morphological characteristics of senescent lungs is the decrease in bronchioles and increased pulmonary alveolar diameter. Previous text has detailed the physiological functions and morphological changes in aging lungs. At the molecular and cellular levels, several aging mechanisms have been proposed by López-Otín et al., including cellular senescence, mitochondrial dysfunction, immunosenescence, and homeostatic disruption, which act on the pulmonary epithelium, impairing its repair function, resulting in loss of “fidelity”, and manifesting in related pathological findings such as fibrosis and airway wall remodeling. These have been demonstrated in diseases such as COPD, idiopathic pulmonary fibrosis (IPF), and acute respiratory distress syndrome (ARDS) (88–91). It should be noted that although cell senescence is related to disease, it is also a normal life activity of the normal lung tissue to maintain homeostasis (92, 93). Next, we will explore how these aging mechanisms causally contribute to pulmonary diseases and identify potential therapeutic targets.

### Aging and cell senescence

Aging leads to declines in body function, with notable impacts on lung elasticity and function due to increased stiffness and tissue composition changes (94). At the cellular level, aging is characterized by reduced cell function, cell cycle arrest (mediated by proteins like Cdkn2a and Cdkn1a), or increased apoptosis, connecting subcellular damage such as protein homeostasis disruption and mitochondrial damage to organ aging (95). Specifically, in lungs, aging impairs AEC2 cells, crucial for organ function, by hindering the differentiation of pulmonary epithelial progenitor cells, weakening defense and immune clearance, for example, via HLA-E inhibition (96). The risk of respiratory diseases increases with senescent cell accumulation, with older mice showing more severe lung damage and slower recovery than younger ones (97–99). Senescent cells, although non-replicating, release the senescence-associated secretory phenotype (SASP)—a cocktail of cytokines, growth factors, and enzymes—which plays roles in wound healing, immune response, and aged cell clearance (100). These factors can activate surface receptors like TNFR and ILR, triggering intracellular signaling pathways and activating NF-κB to regulate inflammation and the cell cycle through NEMO, dependent on ATM phosphorylation (101).

Cellular activities significantly depend on age, making the study of cell aging vital for disease understanding and prevention. Despite considerable progress in cell aging research, its mechanisms remain complex and variable, necessitating further in-depth exploration.

### Mitochondrial dysfunction

As cells age, mitochondria experience increased volume, loss of cristae, and inner membrane damage (102–104). Aging disrupts protein homeostasis in mitochondria, damages mitochondrial DNA (mtDNA), and leads to the formation of superoxide-generating electron transport chains (105–107). These changes activate inflammatory pathways like NF-κB, causing inflammation and impairing mitochondria’s ability to manage energy metabolism and cell death regulation. This mitochondrial dysfunction is linked to diseases such as IPF, COPD, and severe asthma, with increased damaged mtDNA found in lung tissues of these patients (102, 108–111). Simultaneously, studies indicate that mitochondrial dysfunction contributes to aging, suggesting a cyclical relationship (65, 66).

### Inflammation and aging

Inflammation has progressed to a chronic state due to lifestyle and biological factors in aging, involving the accumulation of “metabolic waste” triggering inflammation. Misfolded proteins and cell debris activate immune responses by binding to PRRs. Chronic stimuli in aging lungs cause sustained inflammation, leading to tissue damage and an imbalance between pro- and anti-inflammatory actions. Neutrophils release factors like IL, TNF-α, and IFN, increasing systemic pro-inflammatory cytokines and oxidative stress (112). Targeting TNF-α in mice can speed up aging and inflammation. While some inflammation is crucial for fighting pathogens, an excess can damage lung tissue and lead to diseases. Neutrophils release NETs, which have both antibacterial benefits and immune-regulating effects, but excessive production can worsen COPD (73).

It has been found that the intestinal microbiota of elderly people also undergoes certain changes with the body’s inflammatory response (113). Ecological imbalance in the elderly, marked by a shift from anti-inflammatory to pro-inflammatory microbial products in the gut, contributes to inflammation. Additionally, aging lung and adipose cells release SASP, further intensifying inflammation and its associated damage, linking adipose tissue dysfunction with systemic inflammation and aging (114).

### Immunosenescence

Immunosenescence leads to a slow yet prolonged immune response, especially in aging T and B cells, lowering resistance to infections and cancer. Aging diminishes AT2 cells’ renewal and differentiation, weakening immune functions. Aged lungs have fewer effective macrophages in phagocytosis, chemotaxis, and antigen presentation. Single-cell sequencing shows that in IPF patients, aged macrophages come from circulating monocytes, not from lung progenitor cells (115). Puchta et al. found that the senescent phenotype of monocytes in elderly mice is linked to the aging bone marrow microenvironment rather than being intrinsic to the cells (55). Adaptive immunity is also compromised in the elderly, with reduced lymphocyte activation, humoral responses, and lower counts of naive T cells and receptors. The balance between Th17 cells, which promote autoimmunity and inflammation, and Treg cells, which suppress these responses and maintain immune homeostasis, is disrupted (80).

Immune aging contributes to age-related lung diseases, reducing resistance to infections, changing throat microbiota, and increasing harmful bacteria. These factors, combined with lower respiratory function, difficulty swallowing, and poor vocal cord coordination, raise pneumonia risk by allowing bacteria into the lower respiratory tract. In severe asthma, COPD, and IPF, macrophage activity and T-cell activation are decreased due to less phagocytosis and lower T-cell CD28+ expression, resulting in immune function decline (116–122).

### Autophagy

Autophagy, critical for cellular cleanup and turnover in lung cells, involves forming autophagosomes to degrade unwanted components, a process regulated by autophagy-related genes (ATGs) and microtubule-associated proteins (e.g., MAP1LC3B) (123). Key regulators, including transcription factor EB (TFEB), transcription factor A (TFAM), and mammalian target of rapamycin (mTOR), influence this pathway. Aging activates mTOR, leading to increased cell proliferation and reduced autophagy (124).

Autophagy’s disruption, particularly with aging, is linked to lung diseases like fibrosis, COPD, PAH, and cancer. Studies indicate that older mice and IPF patients show more significant declines in autophagy and related inflammation markers. The process where epithelial cells transform and migrate, known as EMT, is key in fibrosis. PINK1, a kinase in mitochondria, is crucial for mitophagy and mitochondrial health, impacting lung function (102, 108). The PINK1-PARK2 pathway, crucial for mitophagy, when impaired, increases the risk of pulmonary fibrosis and hastens cell aging. Mitophagy, vital for lung function due to high energy needs, is disrupted in this pathway, worsening conditions like COPD by enhancing cell damage and aging. Targeting autophagy with treatments like mTOR inhibitors, including rapamycin and everolimus, offers new strategies for managing diseases, notably cancer (102, 108, 125).

### Nutrition sensing and metabolism

Nutrient-sensing pathways in lung tissues change with aging, impacting metabolism. AEC2 cells adjust metabolism based on nutrient and energy levels, responding to stress or hypoxia. Disruption of regulatory pathways (HIF2a, AMPK, and mTOR) with aging impairs nutrient sensing (126). Nutrient-sensing pathways in lung tissues change with aging, impacting metabolism. AEC2 cells adjust metabolism based on nutrient and energy levels, responding to stress or hypoxia. Disruption of regulatory pathways (HIF2a, AMPK, and mTOR) with aging impairs nutrient sensing (127–129). Similar changes are also observed in COPD and severe asthma (130–132). Enhanced insulin-IGF-1-mTORC1 signaling also accelerates the aging process, being the major accelerator of aging (4, 133). Targeting nutrition-related pathways, such as inhibiting the insulin-IGF-1-mTORC1 axis, has shown potential in extending lifespan (6).

### Self-DNA

Self-DNA, released from the nucleus or mitochondria due to cell senescence or damage, acts as a DAMP, triggering autocrine and paracrine inflammatory responses through PRR activation, potentially causing tissue damage and inflammatory diseases (134). Elevated free DNA levels in IPF, COPD, and severe asthma patients’ blood and sputum indicate self-DNA’s role in age-related lung conditions (135). Additionally, neutrophil aggregation and heightened NET production in response to stimuli like IL-8 are noted in COPD and severe asthma (136–138). mtDNA, a key self-DNA source, is particularly effective in inducing lung damage/inflammation. Studies have shown that a link exists between increased systemic inflammation and higher free DNA levels in the bloodstream (139–141).

### Oxidative stress

Oxidative stress occurs when cells produce excess reactive oxygen species (ROS), overwhelming antioxidant defenses and causing cellular damage. ROS, including superoxide ions and hydrogen peroxide, are normal metabolic byproducts essential for signaling and defense mechanisms. However, their imbalance can lead to oxidative damage, contributing to lung diseases. Theories linking ROS accumulation with aging, such as the free radical aging theory and mitochondrial aging theory, emphasize the impact of ROS on age-related changes (142, 143). Elevated ROS levels have been associated with respiratory conditions like pulmonary fibrosis and lung cancer (144).

Oxidative stress negatively affects lung diseases like pulmonary hypertension, COPD, and fibrotic lung disease, with the antioxidant NAC known for counteracting free radical-induced mutagenesis (145). This dual nature highlights the importance of a balanced approach in using antioxidants for lung tissue rejuvenation, acknowledging both their potential benefits and risks in promoting tumorigenesis. Given its significant role in lung health, understanding oxidative stress in lung aging is crucial for unraveling the mechanisms behind lung diseases and maintaining lung health.

## Age-related pulmonary disease

### ARDS

ARDS is a severe and urgent form of lung injury that typically occurs after severe trauma, severe infection, or surgery. The pathological findings include injured alveolar capillary barriers, decreased surfactant, formation of hyaline membranes within alveoli, and alveolar collapse, leading to pulmonary edema, inflammatory manifestations, and respiratory distress with hypoxemia (146, 147). In the acute phase, rapid coagulation and overactive inflammation response result in excessive inflammatory response and lung damage and dysfunction. The ability of the body to repair damaged tissues and restore lung function depends on the severity and duration of the disease. Otherwise, excessive fibrosis will lead to pulmonary fibrosis (146).

**Figure 1:**
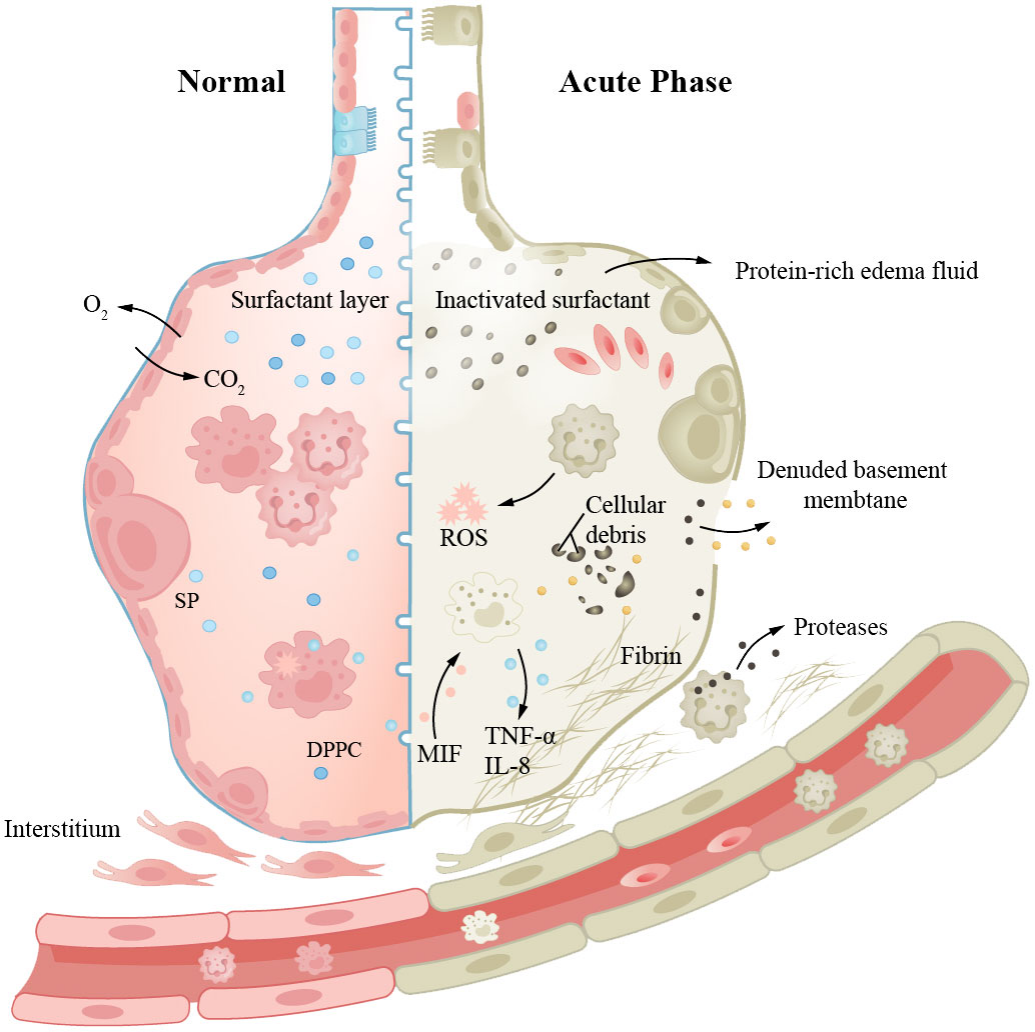
Aging increases the risk of ARDS, and epithelial cell senescence may be a key factor. The left side represents “normal”, while the right side represents “acute injury”. Aging leads to an increased probability of infection, immune dysregulation, and pathological manifestations such as damaged alveolar-capillary barrier, inactivation of surfactant, and formation of intra-alveolar hyaline membranes, resulting in pulmonary edema and inflammatory manifestations. Prolonged disease duration can also lead to pulmonary fibrosis.

Studies have shown that aging increases the risk of ARDS; epithelial cell senescence may be crucial (91, 148). AEC2s senescence, reduced pulmonary stem cell storage, and impaired normal repair may lead to ARDS. In ARDS patients, senescence results in more severe illness and poorer prognosis. In addition, some biological processes associated with ARDS, such as infection, inflammation, and oxidative stress, are also related to aging mechanisms (**Figure 1**). For ARDS patients, mechanical ventilation is usually required to maintain respiration and oxygenation. Ventilation strategies and positive end-expiratory pressure (PEEP) levels need to be chosen according to patient conditions. Severe infection is a high-risk pathogenic factor for ARDS, and it is also a common complication and cause of death following non-infectious ARDS. Aging increases the likelihood of infection, exacerbates immune and inflammatory disorders, and is closely associated with mortality in ARDS patients. Severe SARS-CoV-2 infection may involve long-term pulmonary fibrosis after ARDS; the degree of fibrosis is associated with mortality (149, 150). Antibiotic treatment should be started as early as possible, using broad-spectrum antibiotics and providing sufficient dosage and course. The role of glucocorticoids in ARDS treatment is still controversial, but they are effective in anti-inflammatory and pulmonary fibrosis remission. For ARDS patients, mechanical ventilation is typically required to maintain respiration and oxygenation. The ventilation strategy and PEEP level need to be selected according to the patient’s condition. Prone positioning has been shown to improve gas exchange and respiratory mechanics in ARDS patients (151), but it must be performed with caution to avoid damaging vascular catheters and endotracheal tubes. Studies have shown that ARDS patients usually have lower Health-Related Quality of Lif (HRQoL) scores, and some patients may experience long-term mental health problems, such as anxiety and depression, which will further affect their quality of life. Research on how to enhance patient symptoms and prognosis is ongoing.

### COPD

**Figure 2:**
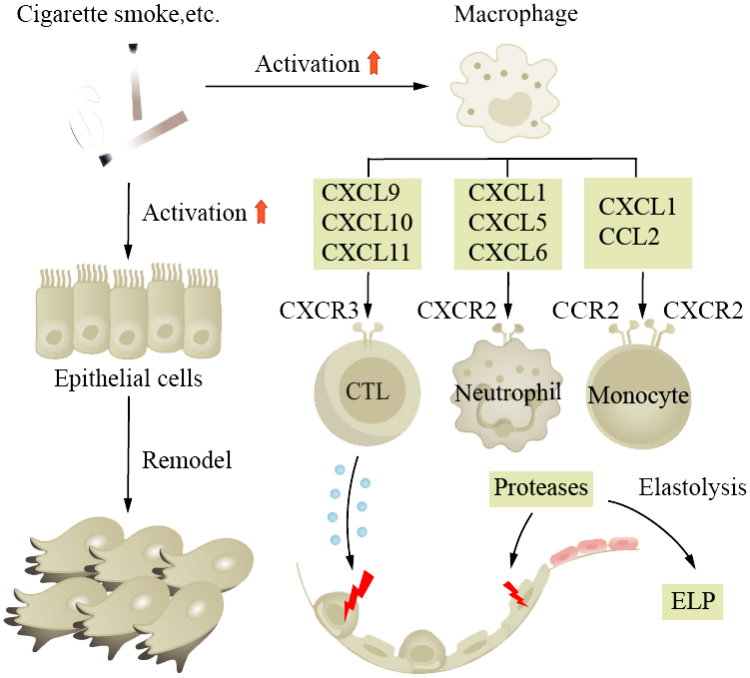
Aging increases the susceptibility to COPD and promotes its progression. Smoking is a major risk factor for the disease, causing long-term inflammation of the small airways, ultimately leading to tissue fibrosis and airway wall remodeling. In aged lungs, the function of airway progenitor cells decreases, resulting in chronic inflammation. Macrophages, under the influence of chemokine CXC subfamily, damage alveolar epithelial cells. ELP, exogenous lipoid pneumonia.

COPD is a common chronic inflammatory lung disease in the elderly. Clinical research indicates that one-fifth of all hospitalized patients aged 75 and older have COPD, highlighting the age-dependent nature of the disease. It is characterized by cough and exertional breathlessness, with irreversible damage to pulmonary function. Smoking, air pollution, and occupational exposure are contributing factors, with smoking being the most important. Patients have long-term inflammation in their small tracts, eventually leading to tissue fibrosis and AWR. We find that in normal aging lungs, the tissue is also in a state of chronic inflammation, similar to the pathological state of COPD patients (152). Airway stem cell (such as club cell) senescence leads to decreased renewal and differentiation functions, resulting in AWR. Age-related changes in respiratory structure and function increase the susceptibility of the elderly to COPD (153–157). Scientists also propose whether the disease triggers change in the baseline level of normal aging lungs (152). Studies have found that there are numerous aging epithelial cells and fibroblasts in patient tissue sections. Fibroblasts isolated from diseased lungs show senescent phenotype and abnormal repair capacity (158). Monocytes derived from circulating blood play a crucial role in pulmonary fibrosis (159), where macrophage phagocytosis activity is decreased, through the action of CXC chemokine subfamily members, interacting with relevant receptors on CTL and monocytes; secretion of corresponding cytokines leads to damage of alveolar epithelial cells (**Figure 2**). The pro-inflammatory cytokines (IL-6 and TNF-α) and MMPs produced by these macrophages are associated with disease severity. Basal cells, as progenitor cells of the airway, have their self-renewal and differentiation abilities impaired after disease onset, leading to delayed wound healing and even abnormal healing (160, 161). Clinical control experiments show that COPD patients secrete more SASP and the secretion increases with age (162). Scientists detected excessive ROS in patient lung tissues and BAL, accompanied by reduced mitochondrial respiration and corresponding increased levels of damaged mtDNA (163). Cilia are cell organs on the surface of airway epithelial cells that can clear mucus and bacteria from the respiratory tract through regular movements. Decreased ciliary clearance function is also an important cause of airway inflammation and infection in COPD patients. Studies have found that the ciliary clearance function in COPD patients is significantly lower than that in healthy individuals, which may lead to bacterial retention and proliferation in the respiratory tract, thereby causing infection and inflammation.

Aging is an independent risk factor for COPD, and various stimuli that promote aging pathways deserve our attention and discussion. Smoke contains a large number of harmful chemicals, such as tar, carbon monoxide, and nicotine, which cause oxidative stress in lung cells, generate free radicals, damage cell DNA, shorten telomeres, and accelerate cell aging. It also triggers inflammation, leading to cell and tissue damage, and ultimately reduces lung elasticity and fibrosis. Emphysema is a chronic lung disease characterized by the destruction of alveolar walls and excessive inflation of alveoli. Smokers are more susceptible to it than non-smokers. Klotho is a b-glucuronidase that has been found to be deficient in the early stage, which may contribute to lung tissue damage and inflammation (164). In smokers, Klotho levels are further reduced, making the lung tissue more sensitive (165).

AECOPD, or the acute exacerbation of COPD, refers to a persistent deterioration beyond the daily situation in a short period of time, 80% of which is caused by bacteria. It is characterized by shortness of breath, increased sputum production, and purulence (11, 166). Common diagnostic methods include bacterial culture and PCR detection. Antibiotics are the mainstay of treatment, and the choice of antibiotics should be based on the pathogen. Traditional Chinese medicine, such as Yupingfeng, is also effective in treating COPD, especially for patients with severe cough and sputum situation (167, 168). Acute exacerbations put COPD patients at risk of pulmonary failure, severe damage to pulmonary function, and decreased quality of life.

### IPF

**Figure 3:**
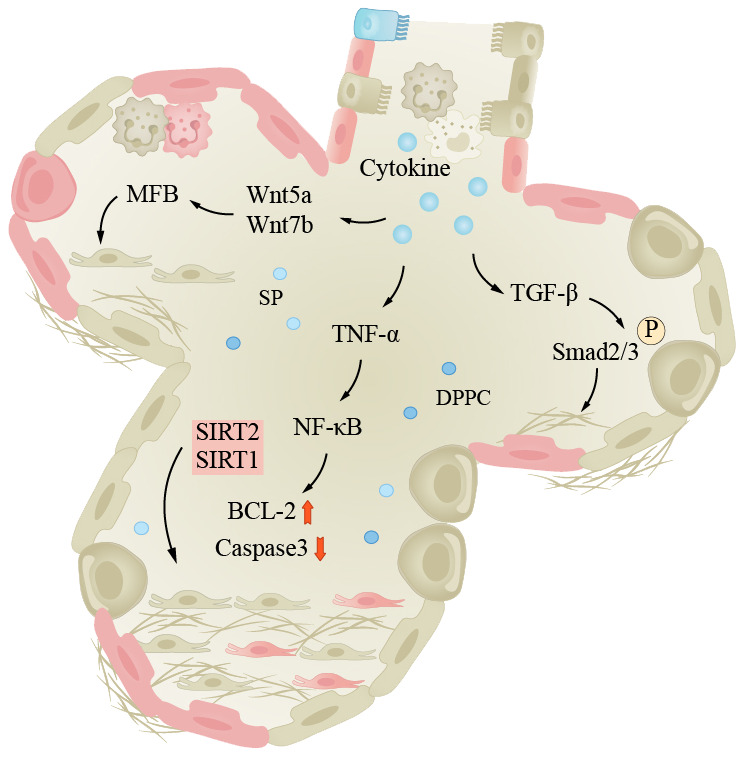
Lung fibrosis under the influence of aging. The pathogenesis of IPF is complex, inflammation is an important process in pulmonary tissue remodeling, and aging is a significant factor in inducing infection. The pathways shown in the figure are important pathways for inflammation-induced pulmonary fibrosis. Smad, Smad protein; TGF-β, transforming growth factor-beta; TNF-α, tumor necrosis factor-α; Wnt/β-catenin, wingless/β-catenin pathway; NF-κB, nuclear factor-κB.

IPF is the most common type of fibrotic interstitial lung disease (ILDs), which will be focused on here. IPF is a progressive interstitial lung disease characterized by progressive breathlessness, coughing, and chest pain. The lung tissue alternates between injury and repair, eventually leading to the formation of large amounts of fibrotic tissue and even scars, reducing pulmonary elasticity and impairing gas exchange function (169). The etiology and pathogenesis of IPF are complex. The specific cause is still unclear; it has been found that adult mouse AEC2s senescence leads to pulmonary fibrosis, which is similar to humans, and fibrosis is associated with p21/p53 and TGF-β. Researchers found that mice exhibited reduced pulmonary fibrosis after treating selective anti-aging AEC2s (170). Currently, there is research evidence indicating that pulmonary fibrosis is associated with high glycolytic behavior. Inflammatory response is a crucial step in initiating lung tissue remodeling, as inflammatory cells release inflammatory cytokines, chemokines, and enzymes that damage alveolar walls and surrounding tissues. Fibroblasts are activated and secrete collagen, elastin, and extracellular matrix components such as proteoglycans, which continue to produce and deposit in damaged areas, eventually forming fibrotic scar tissue. The TGF-β/Smad signaling pathway is considered a key regulatory factor for pulmonary fibrosis (171). TGF-β binds to cell surface receptors, activating the Smad signaling pathway and promoting fibroblast proliferation and collagen synthesis and ECM remodeling. The Wnt/β-catenin signaling pathway also plays a role in the pathogenesis of IPF. Wnt proteins bind to cell surface receptors, leading to the accumulation of β-catenin in the cell nucleus, activating downstream genes and causing pulmonary fibrosis (172). The TNF-α/NF-κB signaling pathway is a classic pathway. In this pathway, TNF-α binds to cell surface receptors, activating the NF-κB (nuclear factor-κB) signaling pathway, leading to inflammation and fibrosis (**Figure 3**). Furthermore, patients with pulmonary fibrosis may exhibit mtDNA damage, manifesting premature aging symptoms and inflammatory responses (163).

The repair capacity of aged lungs decreases, and the repair process does not end with the removal of fibroblasts, but rather produces a continuous fibrotic response, which is associated with a positive feedback of fibroblast apoptosis inhibition. This positive feedback refers to the ability of fibroblasts to produce signals that inhibit their own apoptosis, thus reducing or preventing their own death (173, 174). In some chronic inflammatory lung diseases, fibroblasts can produce various growth factors and cytokines, such as TGF-β, epidermal growth factor (EGF), and IL-6, which promote fibroblast proliferation and migration and inhibit their apoptosis. Clinical studies have shown that those who survive acute diseases are often affected by long-term lung damage and reduced HRQoL. IPF patients usually show earlier aging-related physiological changes than healthy individuals. Moreover, researchers have found that aging-related genes (SIRT1, SIRT2, and FOXO3) and proteins (β-galactosidase) may play a role in the pathogenesis of IPF. Although the connection between age and IPF is established, the interplay between other contributing factors and age remains unknown. Further investigation is required to elucidate the specific signaling pathways and molecular interactions involved in the regulation of these processes. Genetic factors also contribute to the disease; mutations can make individuals more susceptible to pulmonary fibrosis. Environmental factors, such as smoking and occupational exposure, may also affect the incidence of IPF.

### Pneumonia

**Figure 4:**
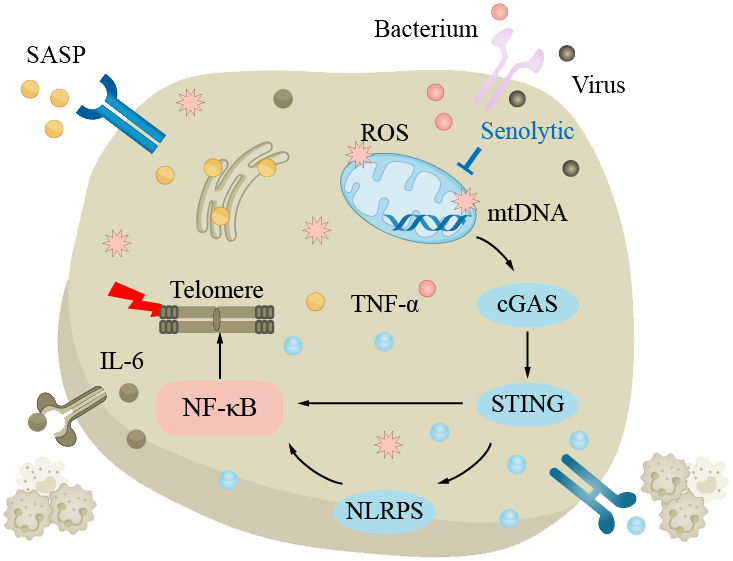
The clearance mechanism of the lungs in the elderly is impaired, which increases the susceptibility to pneumonia. In the elderly lungs, excessive bacteria accumulate in the lungs, making pneumonia more likely to occur. The decline in lung function with aging is attributed to elevated levels of reactive oxygen species (ROS) and impaired mitochondrial function, resulting in telomere damage and upregulation of senescence-associated secretory phenotype (SASP) via the NF-κB pathway, triggering a series of subsequent reactions that increase the risk of pneumonia.

Pneumonia is a pulmonary inflammation caused by bacteria, viruses, fungi, or parasites. The common pathogenic bacteria of bacterial pneumonia are pneumococcal bacteria, which are the most common cause of community-acquired pneumonia (CAP) in the elderly, mainly affecting the pulmonary parenchyma (175). The main pathogenic viruses of viral pneumonia are influenza viruses and coronaviruses, mainly affecting the pulmonary interstitium. The main symptoms of pneumonia include coughing, fever, shortness of breath, and chest pain. According to World Health Organization data, CAP is the most common type of pneumonia; the mortality rate of pneumonia is approximately 12% in developing countries and approximately 9% in developed countries (176). Generally, the mortality rate of ordinary pneumonia is low, while severe pneumonia and infection with viruses such as COVID-19 have higher mortality rates. Age is a risk factor that increases the elderly’s susceptibility to pneumonia. The elderly have weakened lungs in clearing pathogens, with impaired ciliated epithelium clearance in the airways and swallowing clearance mechanisms, increasing the risk of pneumonia, excessive bacterial adhesion and accumulation in the lungs can easily lead to community-acquired pneumonia (37, 175, 177–180). With increasing age, the body’s immune regulatory function decreases, and the risk of pulmonary infection increases. In elderly chronic pulmonary inflammation, TNF-α can induce epithelial cells to express more TNF receptors and enhance the inflammatory response. In addition, oxidative stress can also lead to the upregulation of epithelial cell surface receptors, such as peroxisome proliferator-activated receptor (PPAR). At the same time, the lung function decline in the aging process and damage to mitochondria can lead to telomere damage and increased SASP through the NF-κB pathway, triggering a series of subsequent reactions, increasing the risk of pneumonia (**Figure 4**). The main treatment methods for pneumonia are anti-infection and symptomatic treatment. When the disease is critical, broad-spectrum antibiotics should be used first to cover all possible pathogenic microorganisms as much as possible.

### Asthma

**Figure 5:**
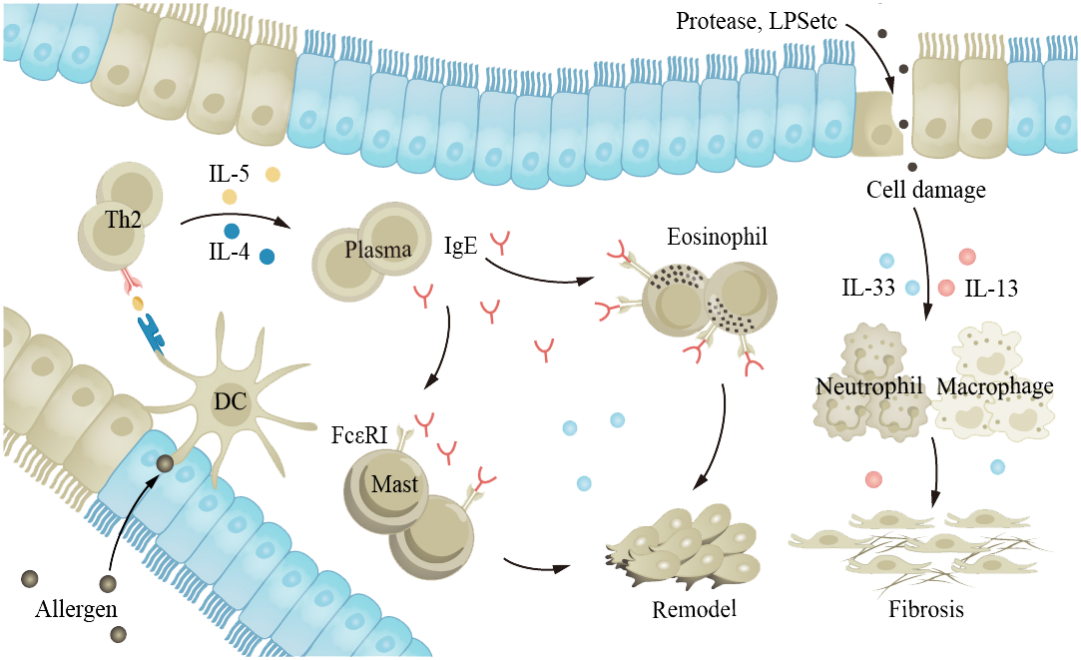
The aging lung is more susceptible to allergens and irritants, exacerbating asthma. Allergens and proteases stimulate the airway epithelium, leading to interactions between various cytokines and receptors within the body, promoting inflammation and causing airway constriction. EGF and FGF play a role in the process of airway remodeling in asthma. FGF, fibroblast growth factor; EGF, epidermal growth factor.

Asthma is a common chronic inflammatory disease of the airways that can occur at any age. The main characteristics of the disease are hyperreactivity of the airways and airway obstruction. Stimuli such as allergens and cold air trigger excessive reactions in the body’s various cytokines and receptors, leading to airway spasm and contraction, and patients experience symptoms such as wheezing, shortness of breath, and coughing. Long-term asthma patients may see an increase in the number of ASM cells, goblet cells, and mucus glands, leading to AWR (39, 181). Although asthma can occur in all age groups, the inflammation and clinical manifestations of asthma in older adults are different from those in younger adults. Studies have shown that severe asthma phenotypes are more common in older adults (182). Older adults have decreased pulmonary immune capacity, making them more sensitive to allergens and stimuli and therefore more susceptible to exacerbation of asthma (15, 60, 62, 64, 183). Th2 secrete IL-4, IL-5, and other cytokines, inducing the production of immunoglobulin E (IgE) and promoting inflammation. IgE is an important antibody in asthma; its main function is to bind to the FcϵRI receptor on the surface of mast cells and eosinophils, leading to cell activation and the release of inflammatory mediators (**Figure 5**). Cholinergic M receptors and histamine H1 receptors are also present in ASM cells and mast cells, and activation leads to ASM contraction. The exact cause of AWR is not clear, but it is known that EGF and fibroblast growth factor (FGF) play a role in asthma AWR.

### Lung cancer

Cancer is a disease characterized by abnormal proliferation and differentiation of cells, usually caused by gene mutations and expression disorders. Age and genetics can increase the risk of developing cancer. The latest statistical data from the National Cancer Center shows that approximately 4.06 million new cases of malignant tumors are diagnosed in China each year, with lung cancer having the highest incidence and mortality rates among malignant tumors, far exceeding those of colorectal cancer, liver cancer, gastric cancer, and breast cancer (184). Lung cancer is also one of the high-incidence and high-mortality malignant tumors globally, with a 5-year relative overall survival rate of approximately 22% (185). It can be divided into squamous cell carcinoma, adenocarcinoma, large-cell carcinoma, and non-small cell lung cancer (NSCLC), which accounts for approximately 80%–85% of all lung cancers. The occurrence and development of NSCLC are closely related to the expression of cancer driver factor, which include gene mutations, amplification, and abnormal expression. Ultra-deep sequencing of normal human skin and esophageal tissue shows that high levels of somatic mutations exist in normal human tissues (186, 187). These mutations are related to skin squamous cell carcinoma and age-related mutations, suggesting that age-dependent microenvironmental changes in the lung play a key role in the progression of lung cancer.

Currently, known oncogenic drivers include epidermal growth factor receptor (EGFR), anaplastic lymphoma kinase (ALK), ROS proto-oncogene 1 (ROS1), B-Raf proto-oncogene (BRAF), and human epidermal growth factor receptor 2 (HER2). The expression of these oncogenic drivers varies in different subgroups of non-small-cell lung cancer patients. Research has found an interesting phenomenon: in lung cancer patients under 50 years old, there is a higher proportion of lung cancer with targetable genomic changes, such as EGFR mutations, ALK or ROS1 fusions, or ERBB2 insertions. In older lung cancer patients, the proportion of other oncogenic drivers, such as KRAS mutations, BRAF V600E, and MET exon 14 skipping, is higher (62, 188). Therefore, we can target different oncogenic drivers and choose corresponding targeted therapy drugs to inhibit their activity, to achieve the purpose of treating NSCLC. With age, DNA damage and mutations in the human body may increase, increasing the risk of cancer (189). At the same time, some genetic mutations related to cancer may also be associated with aging. Genetic factors also play a promoting role in the occurrence of cancer, but it needs to be emphasized that cancer is not inherited directly, but the susceptibility to cancer is inherited, not the cancer itself. Approximately one-eight of cancers are related to genetic gene mutations, while more are influenced by diet, environment, and lifestyle habits. The immune system in elderly lung cancer patients plays an important role in the development of the disease. The body can recognize and eliminate abnormal cells through immune surveillance; effector B cells produce antibodies to clear cancer cells, but at the same time, they can also cause inflammatory reactions, exacerbating the condition. Inflammatory cells can secrete some cytokines and chemokines, such as TNF-α, IL-1, and IL-8. These factors can attract more immune cells to the inflammatory site, promoting the growth and spread of the tumor. Meanwhile, inflammatory cells can also damage the DNA of lung cancer cells by releasing free radicals, thereby promoting the malignant transformation of lung cancer cells.

**Figure 6:**
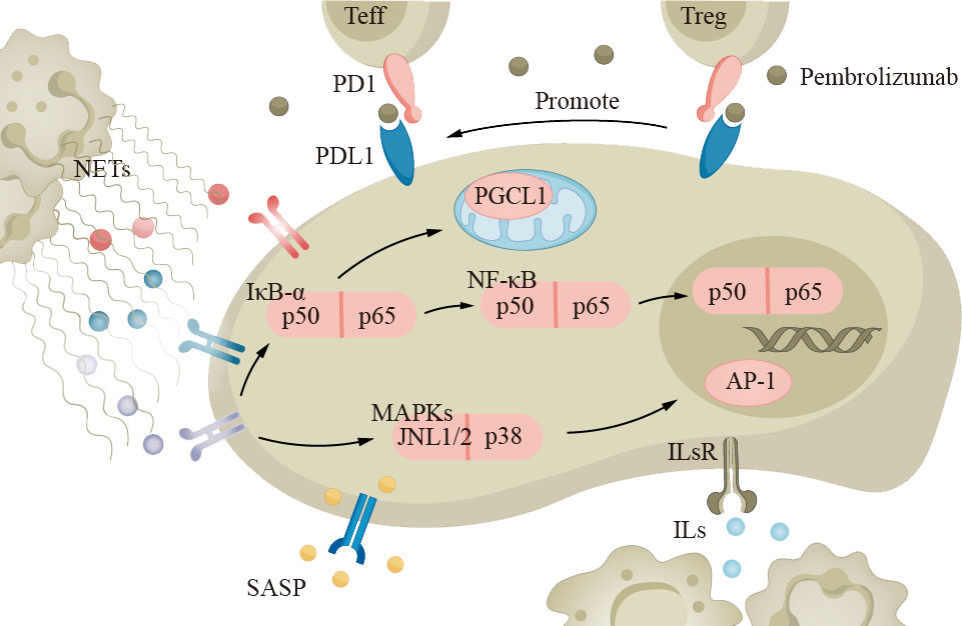
The immune system in elderly lung cancer patients plays an important role in the development of the disease. Neutrophils, macrophages, and other inflammatory cells secrete cytokines and chemokines, such as TNF-α and IL-1, which promote tumor growth and metastasis. Tumor cells can evade the immune system through the PD-1 pathway, inhibiting T-cell activity. Immune checkpoint inhibitors (ICIs) like pembrolizumab can suppress the PD-1 pathway and enhance immune response. ICIs, immune checkpoint inhibitors.

Lung cancer treatment consists of medical and surgical therapies. Chemotherapy, targeted therapy, and immunotherapy are currently common approaches. Immunological checkpoint inhibitors (ICIs) are a type of immunotherapy drug that enhances the immune system’s response by inhibiting checkpoint receptor molecules on immune cells (such as cytotoxic T cells) (**Figure 6**). Examples of targeted PD-1 pathway drugs include pembrolizumab and atezolizumab. Recent studies have shown that the balance of PD-1 expression between effector T cells (Teff) and Treg in the tumor microenvironment can predict the response to PD-1 cancer immunotherapy (63). When the PD-1 on effector T cells binds to PD-L1 on tumor cells, the activity of Teff is inhibited, reducing the ability to attack tumor cells. However, when the PD-1 on Treg binds to PD-L1 on tumor cells, it can enhance the function of regulatory T cells, further suppressing the immune response (190). Although the efficacy of immunotherapy drugs has been reported to decrease, there are currently limited numbers of elderly patients in prospective clinical trials, and we cannot yet draw accurate conclusions (191, 192).

### Sepsis

**Figure 7:**
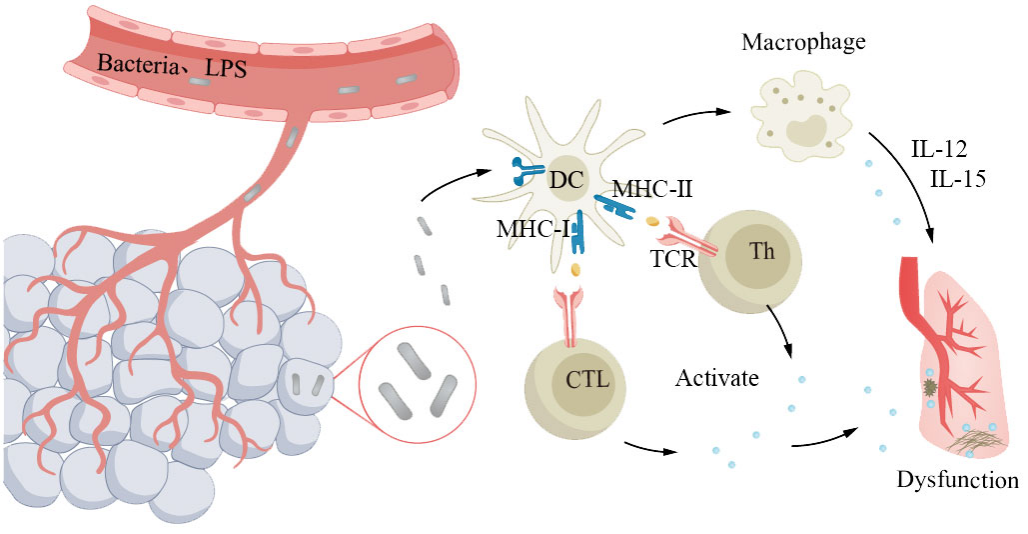
Older adults with abnormal immune function are more susceptible to severe sepsis. The pathogen proliferates locally and invades the bloodstream, leading to systemic symptoms and significant lung damage. Antigens primarily interact with the receptors on antigen-presenting cells expressing major histocompatibility complex molecules and T lymphocytes, triggering the release of cytokines from macrophages and T lymphocytes, resulting in pulmonary dysfunction. Elderly individuals experience a decline in immune function, which makes them susceptible to developing a cytokine storm, leading to exacerbated systemic infection.

Sepsis is a severe systemic infection syndrome characterized by the growth and reproduction of pathogenic bacteria in a local area, continuous invasion of the bloodstream, and production of toxins. These toxins are then disseminated through the bloodstream, causing obvious toxic symptoms and significant damage to other organs and tissues. The most common source of infection in elderly patients with sepsis is the respiratory tract. The underlying cause is the imbalance between pro- and anti-inflammatory responses in the body. Severe sepsis and septic shock are more severe forms of this condition. Due to abnormal immune function, pre-existing diseases, and age factors, the incidence and mortality rates of severe sepsis and infectious shock increase in elderly patients (193, 194). The mortality rate for elderly patients is 50%–60% (194). Cytokine storm is an overwhelming immune response, characterized by a disproportionate production of cytokines, intensifying inflammatory reactions, and leading to systemic infections in elderly patients, which is a key feature in the pathogenesis of sepsis (195). In the course of disease progression, bacterial lipopolysaccharide is the main molecule that induces the production of cytokines. LPS passes through lipopolysaccharide-binding protein, lipopolysaccharide receptor CD14, and Toll-like receptors to activate antigen-presenting cells such as monocytes, macrophages, and DCs, which produce and release cytokines. In addition, exotoxins act as superantigens, bridging the MHC II class molecules expressed on antigen-presenting cells with the receptors on T lymphocytes, promoting the binding of co-stimulatory molecules CD28/CD86, and inducing the production and release of cytokines by macrophages and T lymphocytes (**Figure 7**). With age, the oxidative stress response increases, resulting in an increase in the generation of reactive oxygen species, especially in the aging pulmonary vasculature (196, 197). This leads to more severe clinical symptoms in elderly patients (194, 198). Severe sepsis and septic shock patients often require mechanical ventilation, which is independently associated with increased mortality in elderly patients (194, 198).

## Targeted aging therapy for respiratory diseases

The treatment options for age-related lung diseases are currently limited. Patients with ARDS are often refractory to treatment, and the efficacy of glucocorticoids (GCs) is generally moderate. The mechanism of action primarily involves the binding of the GC receptor (GR) to NF-κB in a process known as “transrepression” (199, 200). NF-κB serves as a central mediator of inflammation and aging, and it represents a potential therapeutic target for age-related lung diseases (201).

COPD or severe asthma patients may experience symptom relief following GC treatment (202). In patients with GC refractory obstructive airway diseases, the use of theophylline and phosphoinositide 3-kinase delta (PI3K-δ) inhibitors can be considered to reduce the acetylation of GR/histones and achieve therapeutic goals (203). Some novel biologic therapies, such as omalizumab and mepolizumab targeting specific pathways, have shown promise in treating severe asthma patients, although individual responses may vary. The ongoing “Targeting Aging with Metformin (TAME)” trial aims to evaluate the health effects of metformin in individuals aged 65–80, as it may reduce the risk of adverse outcomes in asthma and COPD patients.

Anti-aging drugs possess significant therapeutic potential in pulmonary diseases, particularly for IPF patients, through inducing apoptosis. Studies have shown that anti-aging drugs can effectively restore the physical function of IPF patients, often combining dasatinib and quercetin. Dasatinib is a selective tyrosine kinase inhibitor that is commonly believed to mitigate the degree of pulmonary fibrosis and improve patients’ lung function and quality of life, whereas quercetin can inhibit inflammatory responses and fibrotic processes. The combination of both drugs has a synergistic effect, known as the Dasatinib–Quercetin (DQ) mixture. Another therapeutic approach called senomorphics works by intervening in specific mechanisms during the aging process rather than inducing cell apoptosis (204).

Targeted therapeutic strategies that activate DNA via PRRs can mitigate inflammatory responses in age-related pulmonary diseases. By inhaling recombinant DNaseI, high levels of extracellular DNA released by inflammatory cells after pulmonary infection can be degraded, thereby reducing inflammation (205, 206). H-151 is a potent STING inhibitor that achieves its therapeutic effect by inhibiting the cGAS-STING axis. Overall, DNaseI and H-151 exhibit potential therapeutic effects in pulmonary injury and disease models. However, further research is needed to demonstrate their efficacy and determine their potential impact in clinical applications.

Targeted therapy for lung cancer involves treatment strategies aimed at specific molecular targets within lung cancer cells. These targets can include aberrantly active proteins, mutated genes, or overexpressed receptors. By attacking these targets, tumor cells can be targeted more precisely while minimizing damage to normal cells. EGFR is a tyrosine kinase receptor whose aberrant activation or mutation is associated with the development and progression of certain NSCLC. Drugs targeting EGFR include Gefitinib and Erlotinib. ALK gene fusion is common in some NSCLC patients, and drugs targeting ALK include Crizotinib and Alectinib. These drugs inhibit the activity of ALK fusion proteins, blocking tumor cell proliferation. Additionally, previously mentioned PD-1 and PD-L1 immune checkpoint proteins help tumors evade immune attack by inhibiting immune responses in the tumor microenvironment. Targeted drugs include Pembrolizumab and Nivolumab.

We are all aware of the close association between the development of sepsis and the abnormal release of inflammatory mediators. Therefore, some research is exploring treatment approaches that target inflammatory mediators to suppress the inflammatory response. For example, anti-TNF drugs, anti-IL-1 drugs, etc. inhibit the production of inflammatory mediators, thereby reducing the inflammatory response and organ damage. It is also possible to target the modulation of the immune system, specifically by activating co-stimulatory signals in T cells, such as anti-CD28 antibodies, to enhance the immune system’s responsiveness and control infection.

Given that pulmonary diseases can also accelerate aging, targeted therapy against aging mechanisms could provide broad clinical benefits, aiming to prevent pulmonary diseases and complement more specific medical interventions.

## Conclusion

Lung aging is a complex process characterized by cumulative damage and repair changes in the pulmonary cell system. It is closely related to the microenvironment of the lung. Age-related intrinsic mechanisms, such as stem cell pool depletion, mitochondrial dysfunction, increased oxidative stress, and telomere shortening, disrupt the maintenance of pulmonary cell homeostasis. Normal lung aging is associated with various structural and functional changes in the respiratory tract, leading to declines in pulmonary function, lung remodeling, reduced regeneration, and increased susceptibility to pulmonary diseases.

The lung has multiple innate and adaptive defense systems to maintain homeostasis and respond to external stimuli. However, with aging, various cell types in the lung, such as AEC1s, AEC2s, fibroblasts, endothelial cells, and ASM cells, undergo compositional and functional changes, increasing the susceptibility of older adults to the development and progression of pulmonary diseases. Immune senescence exacerbates the production of oxygen-free radicals and increases the production of pro-inflammatory cytokines, making persistent lower respiratory inflammation a reason why older adults are more susceptible to toxic environments and accelerated lung function decline. The poor prognosis and recovery of pulmonary diseases are attributed to age-related changes in innate and adaptive immune responses. Genetic background and lifestyle further promote pulmonary age-related changes, increasing the incidence and progression of airway diseases and susceptibility to infectious stimuli and toxins. Although research has revealed how the immune system of older adults is susceptible to bacterial and viral lung infections, the challenge lies in identifying which age-related molecular changes are targetable and which will have therapeutic benefits. Therefore, to prevent and treat pulmonary aging-related diseases, it is necessary to focus on the changes in the pulmonary microenvironment and take corresponding measures for intervention. In-depth study of lung aging mechanisms, exploration of age-related immune changes, and construction of prevention and immunomodulatory strategies are crucial for improving the prognosis of elderly patients.

## Author contributions

YW: Writing – original draft. XH: Writing – original draft. GL: Writing – original draft. YX: Writing – original draft. XD: Writing – original draft. YL: Writing – original draft. ZW: Writing – original draft. SZ: Writing – original draft. SW: Writing – original draft. HC: Writing – original draft. TT: Writing – original draft. LH: Writing – original draft. LCY: Writing – original draft. LY: Writing – original draft. YC: Writing – original draft. ZJ: Writing – original draft. CH: Writing – review & editing. ZH: Writing – review & editing, Writing – original draft, Supervision, Conceptualization. XZ: Writing – review & editing.
